# Daily Milk Losses Associated with Dairy Cow Bunching, Cattle’s Protective Behavior Against Stable Flies (*Stomoxys calcitrans*) in California

**DOI:** 10.3390/vetsci12111035

**Published:** 2025-10-26

**Authors:** Wagdy R. ElAshmawy, Fernanda C. Ferreira, Deniece R. Williams, Alec C. Gerry, Sharif S. Aly

**Affiliations:** 1 Veterinary Medicine Teaching and Research Center, School of Veterinary Medicine, University of California Davis, Tulare, CA 93274, USA; dvmwilliams@ucdavis.edu (D.R.W.); saly@ucdavis.edu (S.S.A.); 2 Department of Internal Medicine and Infectious Diseases, Faculty of Veterinary Medicine, Cairo University, Giza 12211, Egypt; 3 Clean Air Task Force, Boston, MA 02109, USA; fferreira@catf.us; 4 Department of Entomology, University of California Riverside, Riverside, CA 92521, USA; alec.gerry@ucr.edu; 5 Department of Population Health and Reproduction, School of Veterinary Medicine, University of California Davis, Davis, CA 95616, USA

**Keywords:** economic losses, California, cow bunching, stable flies, milk production, free stall, dairy cattle

## Abstract

**Simple Summary:**

Cow bunching is a seasonal behavioral problem associated with stable fly biting. In response to the painful bites of these flies, cows tend to aggregate in groups, often leaving shade, water, and feed for variable time periods during the day. This behavior impacts milk production. The current study estimated the economic losses associated with cow bunching behavior and stable fly biting activity. This study estimated the highest loss of USD 0.34/cow/week and USD 1.86/cow/week during the last week of May for cow bunching and stable fly attacks, respectively. The lowest loss occurred during the last week of July (USD 0.03/cow/week and USD 0.29/cow/week for cow bunching and stable fly attacks, respectively). The outcomes of the current study will help California dairy producers to budget for their efforts to control stable flies and reduce cow bunching during the bunching season.

**Abstract:**

Cow bunching is a behavioral phenomenon where cattle aggregate in tight groups to protect themselves from biting by stable flies (*Stomoxys calcitrans* L.). The incidence of bunching varies between dairies and even among pens within the same dairy, as it is associated with the location-specific biting intensity of stable flies, which largely varies with dairy management and local environmental factors. Bunching may be associated with decreased feeding and laying times, as well as heat stress due to cattle aggregation. Thus, bunching may affect dairy cows’ milk production by reducing dry matter intake and rumination. To our knowledge, there are no previous studies specifically addressing the effect of cow bunching on milk production in lactating dairy cows. The objectives of our study were to estimate the economic impact of cow bunching against stable flies on milk production on a commercial California dairy and to estimate the economic losses associated with cow bunching and stable fly biting per cow per year. A longitudinal study was conducted from 1 May 2017 through 31 July 2017 on a 5000-cow Holstein herd housed in free stall pens in Tulare County, California. Pen-level cow bunching in four lactating cow pens was recorded weekly for 12 weeks. Bunching observations each day were matched to daily milk records for the study dairy. Two-piece spline linear mixed models were used to estimate the impact of cow bunching and stable fly counts on milk production. Cows in pens where bunching occurred experienced a significant milk reduction of 0.45 kg ± 0.104 (SE) per cow (*p* < 0.01) on the day of bunching in comparison to cows in pens without bunching. There was a significant reduction of 0.6 kg/cow/day in milk production associated with each increase in one stable fly per cow leg (standard metric for recording stable fly biting activity) after adjusting for parity, temperature humidity index (THI), and days in milk (DIM). Based on the economic analysis conducted on weekly bunching and fly counts, modeled milk production losses were reported as weekly loss in milk revenue per cow. The estimated economic loss associated with cow bunching and stable fly counts was highest during the last week of May (USD 0.34/cow/week and USD 1.86/cow/week, respectively) and was lowest during the last week of July (USD 0.03/cow/week and USD 0.29/cow/week, respectively). To mitigate the most substantial economic loss, dairy producers should focus their efforts on controlling stable flies during the early stable fly season, when stable fly abundance tends to be highest.

## 1. Introduction

Stable flies are blood-sucking insects that affect the health, welfare, and productivity of dairy cattle [1,2]. Stable flies tend to bite the lower parts of the body, including the legs and lower abdomen of cattle. To avoid painful bites from these flies, cattle aggregate into a tight group to protect themselves from stable fly attacks, a behavior known as cow bunching [3,4,5]. Cow bunching may protect cattle from stable fly bites through the dilution effect, in which the number of flies per cow decreases as the number of cattle comprising the bunch increases. Cows will compete for position within the bunch as cows in the middle of the bunch experience fewer bites in comparison to cows on the periphery [6,7]. A previous study showed that stable fly attack is associated with increased cortisol levels, indicating stress on the affected cows [1].

The incidence of cow bunching against stable flies varies between dairies and among pens within the same dairy, as it is associated with stable fly biting intensity at the pen level [4]. Stable fly biting intensity varies with fly abundance and fly activity, which are affected by management practices and various environmental factors, including ambient temperature, relative humidity, and the amount of rain in the early spring [8,9,10]. Cows experienced increased odds of bunching when sticky traps captured ≥ 150 flies/trap/week or when lactating cows had biting rates ≥1 fly/front leg [4]. Stable fly activity in California is typically greatest during late spring and early summer, with abundance associated with spring rain and ambient temperature throughout spring and summer [11].

When stable fly biting activity is high, milk production losses in lactating cows can be anticipated due to both direct biting impacts and to cow bunching behavior, increasing cow heat stress and decreasing time spent eating and resting [1,12]. However, only a few studies have evaluated milk production losses relative to stable fly biting rate [13,14,15]. There are no previous estimates specifically for the economic impact of cow bunching on milk production in lactating dairy cows. Freeborn (1928) reported 9.2% milk loss attributed to stable flies [14], and Bruce and Decker 1958 reported a milk loss of 0.65%/fly/cow [15]. Taylor et al. (2012) estimated a median annual loss of 139 kg of milk per dairy cow due to stable flies [16].

The objectives of our study were to (1) estimate milk loss in lactating cattle associated with cow bunching against stable flies, (2) estimate milk loss associated with stable fly biting activity, and (3) estimate the annual economic loss per cow associated with both cow bunching and stable fly biting across five California counties (Fresno, Kern, Kings, Madera, and Tulare), which comprise the top dairy-producing region in the state. The risk factors associated with stable fly activity and cow bunching on California dairies were previously published (El Ashmawy 2019; 2021) [4,17]. The current study expands on earlier research by evaluating the impacts on milk production due to stable fly biting activity and associated cow bunching on one of the study dairies and by extrapolating these losses across five California counties that form a major dairy region of the state. We anticipate this study will help dairy producers evaluate the costs of implementing management practices to reduce stable fly activity or to reduce cow bunching in relation to expected milk losses resulting from the activity of stable flies.

## 2. Materials and Methods

### 2.1. Modeling Milk Production Loss Associated with Cow Bunching and Stable Fly Biting the Current Study Was Approved on 25 February 2016 by the Institutional Animal Care and Use Committee, University of California, Davis (Protocol Number 19088)

#### 2.1.1. Study Design

A previously published longitudinal study on cow bunching and stable fly activity was conducted on 20 dairies in Tulare and Kings Counties (California) between 1 May and 31 July 2017, providing data on stable fly activity and cow bunching [4]. One of these dairies was selected to determine how stable fly activity and cow bunching behavior impact milk production. The selected dairy is a 5000-cow lactating Holstein dairy in Tulare County, CA, containing eight free-stall pens with an exercise area for the lactating cows in each pen. All cows were milked three times per day and fed a total mixed ration (TMR) twice daily.

On the study dairy, stable fly activity and cow bunching were recorded weekly between 9:00 and 11:00 a.m. and again between 12:00 and 2:00 p.m., as previously described [4]. The study definition of cow bunching was that the majority of the cows were aggregating in one location of the pen, and cows in the periphery showed signs of stable fly attack, such as leg stomping, tail switching, and skin twitching. Stable fly activity was recorded using “leg counts” or the number of stable flies on the outside of one front leg and the inside of the other front leg of 15 cows per pen to provide a mean leg count/cow for each pen. Both bunching and leg counts were recorded in four of eight pens representing different locations of the dairy facility. This was done to estimate the impact of bunching and stable fly biting on milk production, given the cow distribution in pens. Ambient temperature and relative humidity were recorded during each count using a mobile application (AccuWeather^®^, American Media Company, State College, PA, USA).

#### 2.1.2. Milk Data

The daily milk production for each cow was calculated by the sum of the milk production for each of the three daily milking times. Data were collected weekly using the weekly backup from Dairy Comp305 @ 2017 VAS (Valley Ag Software, Tulare, CA, USA). For any missing values, we used the mean of the milk production for the previous 7 milkings and the following 7 milkings.

#### 2.1.3. Statistical Analyses

Descriptive Statistics: Data on cow bunching and stable fly counts between 9:00 and 11:00 a.m. and 12:00 and 2:00 p.m., ambient temperature, and relative humidity were recorded weekly. Cow data, including cow ID, parity (1, 2, and ≥3), days in milk (DIM), and milk production for each milking, were obtained from the weekly DairyComp 305 backup.

##### Modeling Milk Production Loss

A two-piece mixed-effects spline regression model was used to model the lactation curve for each cow using a 2-piece linear function for DIM, as described by Aly et al. (2010) [18]. Briefly, the 2-spline function of the model resulted from the shape of the lactation curve, as after calving, there is an initial increase in milk production from calving to the cow’s lactation-specific observed peak (PK), followed by a decrease in milk production to the end of lactation (dry-off), and DIM were transformed to 2 variables: DIM pre-peak (DIMprePK) and DIM post-peak (DIMpostPK). For cows at pre-peak, DIMprePK was calculated as (DIM at test day − DIM at PK)/DIM at PK. For cows at post-peak, DIMpostPK was calculated as (DIM at test day − DIM at PK)/(total DIM − DIM at PK). For each of the study cows, the resulting splines were joined at a knot equivalent to the cow’s lactation peak.

Bunching and stable fly activity were modeled separately. Fixed effects included in the model were bunching (yes/no) or stable fly activity (mean leg count/cow), parity (1, 2, ≥3), month, ambient temperature (AM or PM), relative humidity (AM or PM), and temperature humidity index (THI) was calculated from the record ambient temperature and relative humidity and included in our analysis. DIMprePK and DIMpostPK were forced into all models given their biological and curve-modeling importance. Cow ID, DIMprePK, and DIMpostPK were included in the model as random effect variables. The model predicted $Y_{ij}$ for the milk yield on the jth cow in the ith pen, as shown in Equation (1).

(1) $${Y_{ij}=\beta_{0}+\beta_{1}X_{1}+{t_{i}}^{pen}+}_{Z_{ij}}cow+u_{1}DIMprePK+u_{2}DIMpostPK+e_{ij}$$

where the intercept $\beta_{0}$ is the overall mean milk production at peak; $\beta_{1}X_{1}$ respective fixed effects (Xβ); ${t_{i}}^{pen}$ is the random effect for the pen; $Z_{ij}$ cow is the random cow intercept; $u_{1}$ DIMprePK and $u_{2}$ DIMpostPK are the random slope coefficients for DIM pre-peak and post-peak, respectively; and $e_{ij}$ is the residual error. The best model fit was using the mean morning stable fly count (AM counts), and from here on, stable fly activity will refer to the mean AM counts.

The computer software used for all statistical analyses was Stata 15.1^®^.

### 2.2. Economic Models for Estimating Milk Loss Due to Stable Fly Activity Across Five California Counties

#### 2.2.1. Model Inputs

1. Milk Data.

The distribution of parity in the herds from Fresno, Kern, Kings, Madera, and Tulare counties was obtained from the regional Dairy Herd Improvement Association (DHIA) data processing center (Agritech Analytics, Visalia, CA, USA), as described below. Mean milk production of a lactating cow in the herd (kg per cow per day) was calculated as the weighted average between the daily milk production of each lactation group and the percentage of cows in each group. To estimate the average days at peak and the average days in milk (DIM) of cows in this region during the bunching season, we obtained DHIA lactation records of cow and herd identification, cow breed, calving date, test-day date, and test-day milk yield. Before editing, our dataset contained 7,380,178 lactation records with 7,342,604 lactation records from Holstein cows only. Our simulation models used data from five California counties (Fresno, Kern, Kings, Madera, and Tulare counties, n = 1,814,065 lactation records). In total, we had 9,575,941 test-day data points, with data further limited to test-day information only from cows calving from April 2017 to December 2017 (a total of 1,039,003 data points), because our bunching study, providing the bunching and stable fly activity data, was performed in 2017. Average herd size was estimated by the percentage of lactating and dry cows per herd per day using a total herd size calculated as the sum of the number of lactating and dry cows [19]. We excluded 349 records from herds with less than 50 cows, 4050 records with missing milk yield information, 107 records from cows missing lactation number information, 8053 records from herds with less than 5 test days per year, and 106,742 records from cows with DIM above 300. Our final dataset contained 919,702 test-day records from 177,643 cows from 85 herds across the five counties.

2. Regional Weather Data.

**Figure 1:**
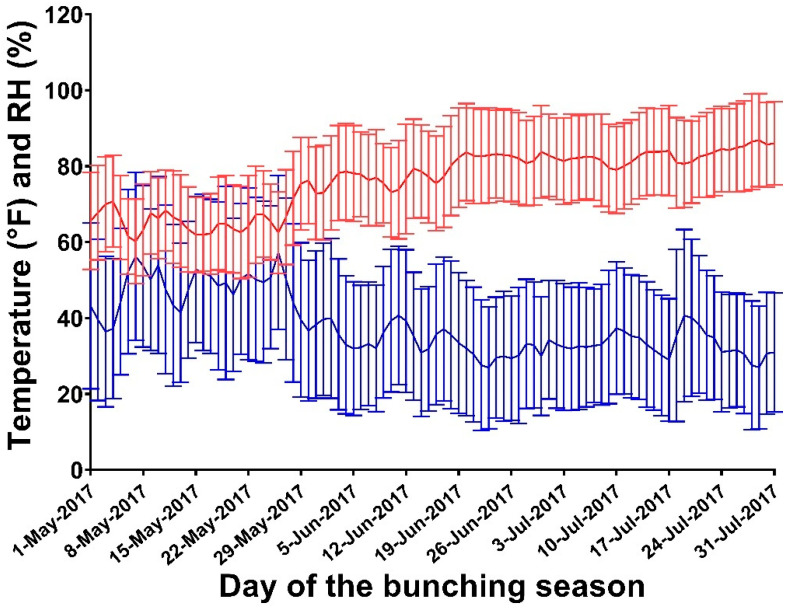
Mean and standard deviations of temperature (red bars) and relative humidity (blue bars) from 2015 to 2019 for the counties of Fresno, Kern, Kings, Madera, and Tulare in the Central Valley of California.

We obtained weather data (average hourly temperature and relative humidity) from the National Oceanic and Atmospheric Administration (NOAA, 2021) weather stations located in the five study counties (Figure 1). These counties are located in the Southern San Joaquin Valley area and share similar weather conditions. We averaged data from all available weather stations within each county for the days between 1 May and 31 July (peak stable fly activity period for the years 2015 to 2019. The THI was calculated according to the formula proposed by Zimbelman et al., 2009 [20]:

THI = (T − (0.55 − (1 − RH))*(T − 58))

where T = average daily temperature (°F) and RH = relative humidity (decimals).

We created four categories of heat stress to be used in the milk production models [20]:

THI < 72, 72 < THI < 78, 78 < THI < 89, and THI > 89.

3. Stable Fly Activity and Associated Cow Bunching.

In this study, stable fly biting activity and cow bunching were recorded weekly for 31 lactating cow pens on 10 California dairies between 1 May and 31 July 2017 during the peak season for stable flies in California. As described earlier, stable fly counts and observation of bunching were recorded twice, between 9:00 and 11:00 a.m. and between 12:00 and 2:00 p.m.

#### 2.2.2. Economic Model

1. Economic impact of bunching on milk production.

**Figure 2:**
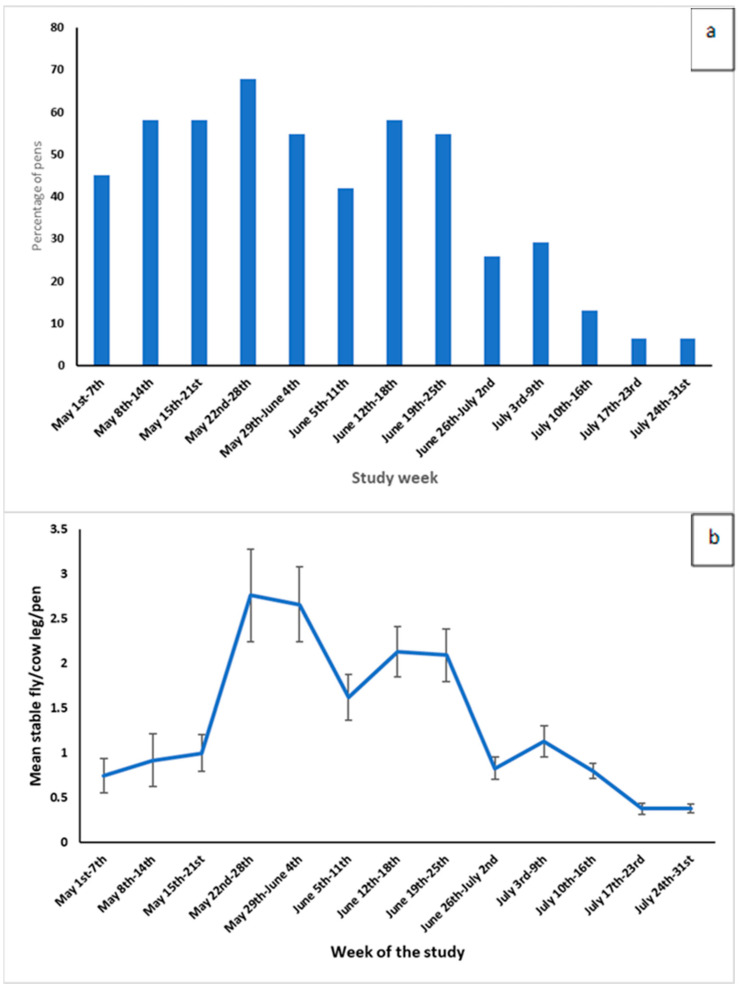
(**a**) The weekly percentage of pens with bunching (**a**) and the mean and standard errors of the number of flies per cow leg (**b**) during weekly observations of 31 lactating cow pens on 10 California dairies between 1 May and 31 July 2017.

An economic simulation model was specified based on the outcomes of the milk loss model due to cow bunching (previously described). For every day of the bunching season, we used the parameters of the milk production model for bunching previously described (Table 1) to estimate the average daily milk production for each lactation group, whether bunching occurred or not. Without bunching, the milk production of an average lactating cow in the herd (kg/lactating cow per day) was calculated as the weighted average between the daily milk production of each production group and the percentage of cows in each group. When bunching occurred, average milk production was calculated similarly; however, we multiplied the estimated milk production by the percentage of pens that bunched and summed it with the milk production from the pens that did not bunch, multiplied by the percentage of pens that did not bunch (1- percent that bunched). This approach allowed us to account for the fact that not all pens showed bunching behavior on each day (Figure 2a). We assumed that the bunching behavior was the same for every day within the same week. We then calculated the difference in milk production per day between cows in farms that did not bunch and cows in farms that did show bunching, accounting for the average percentage of pens that bunched per week.

Economic impact of stable fly activity on milk production.

An economic simulation model was specified based on outcomes of the milk loss model due to stable fly activity (described in the previous section). The parameters of the two-piece spline model of milk loss associated with cow bunching were used in building the economic model (Table 2). The mean number of stable flies per cow leg was used to estimate the daily milk production losses for cows by week throughout the stable fly season (Figure 2b). Briefly, weekly stable fly biting, measured as the mean stable fly count per cow leg, was based on stable fly counts from 31 pens on 10 dairies from Tulare and Kings Counties between May and July 2017, based on work by ElAshmawy et al. (2021) [17].

2. Estimated Days at Peak and Days in Milk during the Bunching Season.

Cows were first grouped according to their lactation number (PRIM, SECON, and THIRD+). To estimate the average days at peak milk yield, we identified the DIM at maximum milk production per lactation group during the study period (1 May to 31 July). Days at peak yield were 92, 60, and 57 for PRIM, SECOND, and THIRD+ cows. The day at peak was used to further split the cows into 6 production groups: PRIM cows with less than or at 92 days in milk (PRIM_PRE), PRIM cows with more than 92 DIM (PRIM_POS), SECON cows with less than or at 60 days in milk (SECON_PRE), SECON cows with more than 60 DIM (SECON_POS), THIRD+ cows with less than or at 57 days in milk (THIRD+_PRE), and PRIM cows with more than 57 DIM (THIRD+_POS). This step was important because our milk production models used the two-piece spline model approach to estimate milk production [18].

To estimate the percentage of cows in each of the six production groups on each day of the bunching season, we first used PROC MEANS (SAS 9.4) to sum the number of cows in our dataset per day for each test day available. Then, we used the approach described by Ferreira et al. (2020) [19] to model the number of cows for each day of the bunching season. For each production group, the number of cows per day was estimated by using a sine–cosine model, with the total number of cows per day as the dependent variable, and the sine and cosine of the day of the year t as independent variables. We used PROC GENMOD (SAS 9.4) to run the model as follows:

$$y_{zt}=\alpha +\beta_{1}sine\frac{2\pi t}{365.25}+\beta_{2}cosine\frac{2\pi t}{365.25}+e_{zt}$$

where $y_{zt}$ is the total number of cows per day *t* for each lactation group *z* in the dataset, *t* is the day of the year (1 to 365), α is the intercept, *β*_1_ and *β*_2_ are the parameters of the sine and cosine terms, and $e_{zt}$ is the residual error. We then calculated the percentage represented by each of the six production groups per day.

To estimate the average DIM of each lactation group in the studied region for each day of the bunching season, accounting for seasonality along the year, we used the same approach described for the total number of cows (Ferreira et al., 2020) [19]. Therefore, for each production group, average daily DIM was estimated by using a sine–cosine model, with DIM as the dependent variable, and the sine and cosine of the day of the year *t* as independent variables. Cow was included as a random effect in the model. We used PROC GLIMMIX (SAS 9.4) to run the models, weighted by the number of observations per day, as follows:

$$y_{izt}=\alpha +\beta_{1}sine\frac{2\pi t}{365.25}+\beta_{2}cosine\frac{2\pi t}{365.25}+{cow}_{i}+e_{izt}$$

where $y_{izt}$ is the test-day milk production per day *t* for each lactating cow i in the lactation group *z* in the dataset, *t* is the day of the year of the test day (1 to 365), α is the intercept, *β*_1_ and *β*_2_ are the parameters of the sine and cosine terms, ${cow}_{i}$ is the random effect, and $e_{izt}$ is the residual error.

Economic Losses. Milk price was calculated as the average milk price received across the US during the previous 5 years (2017–2021) (USD 0.35/kg, USDA-ERS). Feed cost was calculated as the average feed cost per kg of milk produced during the same time period (USD 0.19/kg of milk, USDA-ERS). The marginal milk price was USD 0.16/kg. We assumed a farm with 1000 lactating cows, a calving interval of 395 days, and a dry period of 60 days.

**Figure 3:**
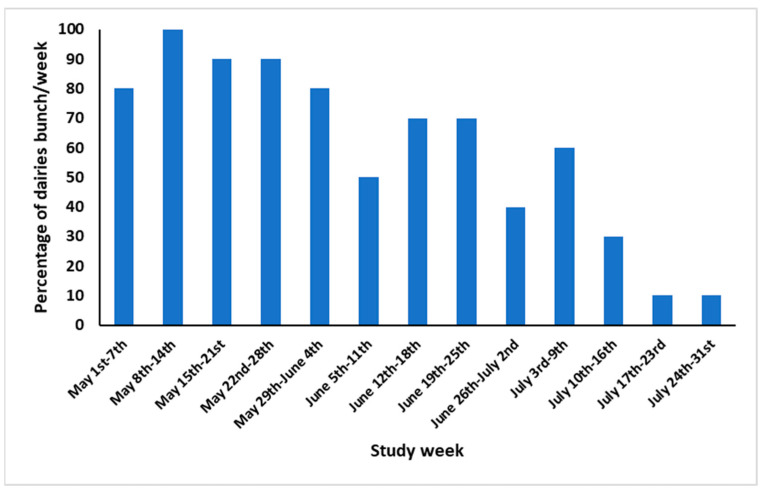
Weekly incidence of cow bunching recorded between 1 May and 31 July 2017 on 10 California dairies.

The difference in milk production (kg/week) between cows on farms that bunched relative to those that did not bunch was multiplied by the marginal milk price. Similarly, the difference in milk production from cows in farms with zero stable flies/cow leg, or farms with a higher mean stable flies/cow leg, was multiplied by the marginal milk price. The milk losses were summarized per week, as the bunching in our study was recorded weekly. To calculate the economic losses for the five study counties, we calculated the weekly percentage of dairies in the region that had bunching events during the bunching season (Figure 3). Using USDA-NASS (2017), we estimated that there were 476 dairies housing 959,245 lactating cows in the five counties (number of dairies/average number of lactating cows per farm, per county: 34/1942 in Madera, 41/2844 in Kern, 65/1581 in Fresno, 101/1717 in Kings, and 235/2129 in Tulare). We then multiplied the number of dairies in each county by the percentage of dairies with bunching events per week and multiplied by the estimated losses per week to obtain the total economic losses due to the drop in milk production during the bunching season.

Stochastic analysis. Differences in the losses associated with cow bunching under varying input values were estimated using stochastic Monte Carlo simulation models using @Risk software (version 8.2, Palisade Corp., Ithaca, NY, USA). Simulations were run and recorded for 10,000 iterations with replacement. To run these models, the variables temperature, relative humidity, milk price, feed cost, and the percentage of pens that bunched per farm within each week were considered stochastic, i.e., an estimated variation was introduced for every iteration. The distributions used for each variable are presented in Table 3.

## 3. Results

### 3.1. Milk Loss Associated with Cow Bunching and Stable Fly Activity on a California Dairy

#### 3.1.1. Descriptive Statistics

The milk production and bunching behavior were recorded for 1073 cows that spent at least 9 weeks in the study from 3 May until 26 July, with a total of 12,262 observations. Cows were divided according to their parity into first lactation (451 cows), second lactation (323 cows), and third lactation or greater (299 cows). The mean (±SE) milk production was 34.90 kg/day (±0.097) for the first lactation heifers, 42.33 kg/day (±0.186) for second lactation, and 43.57 kg/day (±0.205) for cows of third lactation or greater.

#### 3.1.2. Association Between Cow Bunching and Milk Production

Results of the mixed-effects regression model on the association between cow bunching and milk production are presented in Table 1. Cows in pens with bunching were associated with a significant reduction in milk production of 0.45 kg/cow/day (*p*-value < 0.01) in comparison to cows in pens without bunching after accounting for parity, month, THI, and DIM. Multiparous cows had a significantly higher daily milk production (5.07 kg for lactation 2 and 7.25 kg for lactation ≥ 3, *p*-value < 0.01) in comparison to first-lactation heifers. Cows produce significantly more milk, 1.09 kg/day in May and 1.72 kg/day in June, in comparison to July (*p*-value < 0.01). THI > 78 was significantly associated with lower daily milk yield (−0.69 kg when THI >78–89 and −4.70 kg when THI > 89–98) in comparison to THI < 72, while there was a significantly higher daily milk yield of 0.92 kg when THI was between 72 and 78 in comparison to THI < 72 (*p*-value < 0.01).

#### 3.1.3. Association Between Stable Fly Activity and Milk Production

Results of the mixed-effects regression model on the association between stable fly activity and milk production are presented in Table 2. Mean morning (9:00–11:00 am) stable fly activity of one stable fly/cow leg was associated with a significant reduction in milk production of 0.6 kg/cow/day (*p*-value < 0.01) after accounting for parity, month, THI, and DIM. Multiparous cows had a significantly higher daily milk production (6.32 kg for lactation 2 and 7.70 kg for lactation ≥ 3, *p*-value < 0.01) in comparison to first-lactation heifers. Cows produce significantly higher milk, 3.12 kg/day in May and 2.50 kg/day in June, in comparison to July (*p*-value < 0.01). The temperature humidity index was significantly associated with lower daily milk yield (- 0.80 kg when THI > 78–89 and −3.38 kg when THI > 89–98) in comparison to THI < 72, while there was a significantly greater daily milk yield of 1.66 kg when THI was between 72 and 78 in comparison to <72 (*p*-value < 0.01).

### 3.2. Results of the Simulation Model for the Economic Impact of Cow Bunching and Stable Fly on Milk Production for Five California Counties

#### 3.2.1. Incidence of Cow Bunching

The results of the weekly incidence of cow bunching at the dairy level were reported on 31 lactating cow pens on 10 dairies between 1 May and 31 July 2017, as shown in Figure 3. The highest monthly incidence of cow bunching at the dairy level during 2017 was in May (90%), followed by June (62%), and the lowest recorded was in July (27.5%). The weekly incidence of cow bunching at the dairy level showed an increasing trend in May, with all dairies reporting bunching in at least one pen in the second week. It then gradually decreased to the lowest level (10%) during the last two weeks of July. The incidence of cow bunching at the pen level in 2017 is presented in Figure 2a. The monthly incidence of cow bunching at the pen level follows the same trend reported at the dairy level, with weekly incidence of cow bunching on the 31 lactating cow pens increasing in May from 45.2% in the first week to 67.7% toward the end of May then decreasing in June to reach the lowest incidence in the last two weeks of July (6.5%).

#### 3.2.2. Stable Fly Activity

The stable fly activity per cow leg on 31 lactating cow pens on 10 dairies in California between 1 May and 31 July 2017 is presented in Figure 2b. The mean stable fly activity varied among different weeks and months, with an increasing trend in May, reaching the maximum in the fourth week of May (2.76 ± 0.520 fly/cow leg), and the lowest count was recorded toward the end of July (0.38 ± 0.047 fly/cow leg). The minimum recoded count of stable flies was zero, and the maximum was 11.67 flies/cow leg.

#### 3.2.3. Milk Losses Due to Bunching

**Figure 4:**
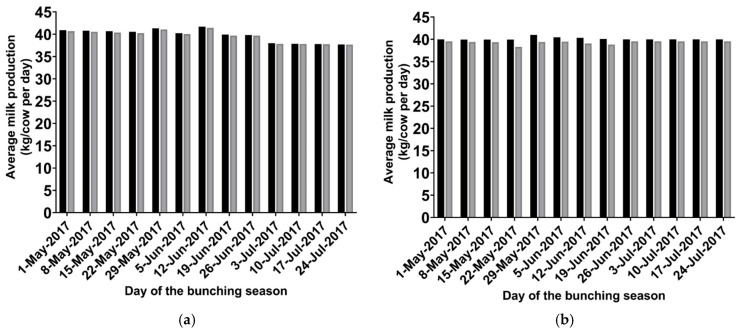
Average milk yield (kg/lactating cow per day) according to the week of the bunching season for (**a**) farms that experienced or did not experience bunching (black bars: averages for cows in herds with no bunching. Gray bars: average for cows in herds experiencing bunching). (**b**) Average milk yield (kg/lactating cow per day) according to the week of the bunching season for cows in farms with or without stable flies (black bars: averages for cows in farms with no stable flies. Gray bars: average for cows in farms with stable flies).

Overall, the average milk produced per cow per day in herds that did not bunch was 39.4 kg, versus 39.2 kg for cows in herds experiencing bunching, from 1 May to 31 July (bunching season) (Figure 4a). The losses varied according to the week due to the percentage of dairies and pens that bunched within that week (Figure 2a and Figure 3). For instance, in the fourth week of the bunching season (started on 22 May), the difference in average milk production per cow per day for herds with no bunching and herds with bunching was 0.305 kg (40.5 kg versus 40.2 kg, respectively), as bunching was observed in 90% of the dairies and 68% of the lactating pens. On the other hand, this difference was only 0.029 kg in the week starting on July 24, when only 10% of the dairies and 6.5% of the pens showed bunching behavior. Overall, during the bunching season, a 1000-lactating-cow farm that experienced bunching events lost a total of 16,389 kg of milk. When we compared only the milk produced by cows in pens that did not bunch versus the milk produced by cows in pens that bunched, the difference was, on average, 0.45 kg/cow per day (40.00 kg versus 39.55 kg, respectively).

#### 3.2.4. Economic Losses Due to Bunching

**Figure 5:**
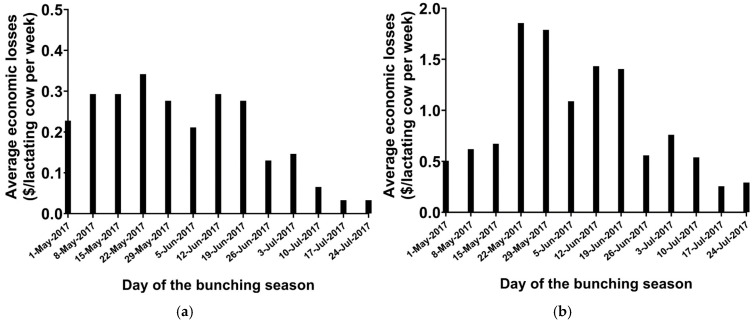
Average economic losses due to bunching (USD/lactating cow per week) according to the week of the bunching season. (**a**) Losses due to bunching and (**b**) losses due to stable fly activity.

**Figure 6:**
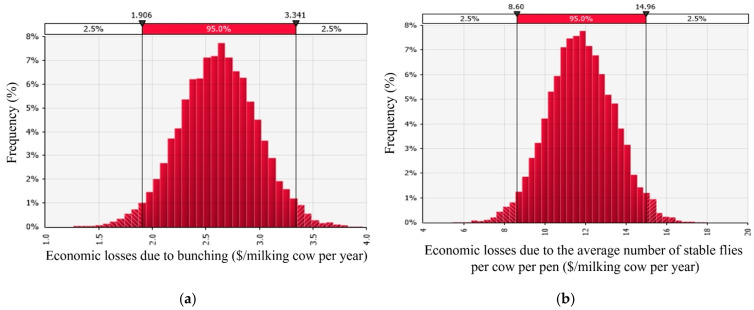
Stochastic analysis of the economic losses (USD/lactating cow per year) due to (**a**) bunching and (**b**) stable fly biting activity during the bunching season in California.

The average economic losses (USD/cow per week) due to bunching are summarized in Figure 5a. The economic losses following milk losses varied according to the week of the bunching season. For instance, in the week starting on 22 May, the average loss was USD 0.34/cow per week, whereas in the last week of the bunching season starting on 24 July, the losses were only $0.03/cow per week. The total loss per lactating cow per year due to the occurrence of bunching behavior in an average farm in the Central Valley of California was USD 2.62. For a 1000-cow dairy, the annual losses amount to USD 2618. For the five counties studied, the average total economic losses during the bunching season were USD 1,857,809/year. Stochastic analysis for economic losses due to bunching under varying temperatures, relative humidity, milk price, feed cost, and the percentage of pens that bunched per farm is presented in Figure 6a. The economic losses ranged from USD 1.35 to USD 4.06/lactating cow per year, with a mean difference of USD 2.62 (95% CI: USD 1.91 to USD 3.34), as shown in Figure 6a. Milk price was the main contributor to the total variance, and it explained 81% of the total variation in the economic losses per cow per year, followed by feed price, which explained 19% of the total variance. An increase in milk price leads to greater economic losses.

#### 3.2.5. Milk Losses Due to Stable Fly Activity

Overall, the average milk produced per cow per day for cows in pens without stable flies was 39.1 kg versus 38.3 kg for cows in pens with stable flies, from 1 May to 31 July (bunching season) (Figure 4b). The milk losses varied weekly due to the variation in the stable fly activity (Figure 2b). For comparison, in the fourth week of the bunching season (started on 22 May), when the mean number of stable flies per cow leg peaked at 2.76, the difference in average milk production per cow per day for cows in pens with a mean of zero stable flies/cow leg versus cows in pens with a mean of 2.76 stable flies/cow leg is 1.6 kg (39.9 kg versus 38.3 kg, respectively). As the stable fly season progressed, the mean number of stable flies per cow leg decreased, and on the week starting on July 24, for instance, the difference in milk production for cows in pens without or with stable flies was only 0.22 kg (37.5 versus 37.3, respectively). On this last week of the bunching season, the mean count of stable flies per cow per pen was only 0.38 flies/cow leg.

#### 3.2.6. Economic Losses Due to Stable Flies

The average economic losses (USD /cow per week) due to the presence of stable flies are summarized in Figure 5b. The economic losses followed milk loss due to the bunching behavior and varied according to the week of the bunching season. For instance, in the week starting on 22 May, the average loss was USD 1.86/cow per week, whereas in the last week of the bunching season starting on 24 July, the losses were USD 0.29/cow per week. The total loss per lactating cow per year due to stable fly attack in an average farm in the Central Valley of California was USD 11.77. For a 1000-cow dairy, the losses per year amount to USD 11,765/year. Stochastic analysis of the economic losses due to the mean number of stable flies per cow per pen under varying temperatures, relative humidity, milk price, feed cost, and the mean number of stable flies per cow per pen are presented in Figure 6b. The economic losses ranged from USD 5.41 to USD 18.00/lactating cow per year, with a mean difference of USD 11.76 (95% CI: USD 8.59 to USD 14.56), as shown in Figure 6b. Milk price was the main contributor to the total variance, and it explained 81% of the total variation in the economic losses per cow per year, followed by feed price, which explained 19% of the total variance.

## 4. Discussion

Cow bunching is a seasonal behavioral phenomenon associated with increased biting activity of stable flies and further associated with management factors on dairy farms [12]. Stable flies are blood-sucking insects that commonly cause painful bites on the lower parts of the body, and cows tend to protect themselves by exhibiting a bunching behavior [2,4,21]. Few studies have estimated the economic impact of stable flies on cattle [13,14,15,16]. To the best of our knowledge, the current study is the first to estimate the effect of cow bunching on milk production of lactating dairy cows. Cow bunching against stable flies was associated with a reduction in milk production of lactating Holstein dairy cows during the stable fly season (May through July) after accounting for the environmental factors (ambient temperature and relative humidity) and cow factors (parity and days in milk). The current study simulated the economic loss in milk production associated with cow bunching and stable flies in five California counties.

### 4.1. The Economic Impact of Cow Bunching on Milk Production

The current study showed a significant reduction in the daily milk production per cow (0.45 kg/cow/day) when bunching occurred after adjusting for parity, THI, study month, and DIM. This reduction could be attributed to the cow’s physiological and behavioral responses to biting stable flies, including fly-repelling behaviors such as tail switching and foot stomping, and aggregation (bunching behavior) as cows compete to be in the middle, as cows at the center experience fewer stable fly bites than cows on the periphery [2,4,21]. Cow bunching may affect dry matter intake and lying time, which is associated with an increase in the number of stable flies in cow pens [1,12]. The loss in milk production per cow during the bunching season varied from 0.305 kg/cow/day at the peak of the season to the minimum of 0.029 kg/day at the end of the bunching season with an average of 0.20 kg/cow/day after accounting for the variability in cow bunching among dairies and the percentage of pens with bunching within the dairy during the bunching season (May through July). The highest percentage of dairies with cow bunching was reported during May, with a maximum during the second week (all dairies had bunching), and the lowest was during July, with the minimum occurring during the last two weeks (5% of the dairies) [4].

Dairy herds with cow bunching produced, on average, 39.2 kg milk/cow/day in comparison to 39.4 kg/cow/day for herds without bunching during the study period. The economic loss varied by the study month, with the highest loss estimated during the last week of May, USD 0.34/cow/week, and the lowest was during the last week of July, USD 0.03/cow/week. The estimated total loss per cow per year across the five California counties varied between USD 1.35 and USD 4.06, with an average of USD 2.62. For a 1000-cow dairy, the estimated average milk losses due to cow bunching were, on average, 200 kg/day during the bunching season (a minimum of 0.03 kg/cow/day to a maximum of 0.31 kg/cow/day). The losses after accounting for the milk price and feed price were USD 2618 per year. The losses reported in the current study may be underestimated, as the study dairies used different fly control measures that may have influenced the incidence of cow bunching. The variation in the economic losses is attributed to the variation in the proportion of dairies and pens that experience bunching, and could be different in other regions due to the variation in the incidence of bunching, milk, and feed price.

### 4.2. The Economic Impact of Stable Flies on Milk Production

The current study showed a significant reduction in the daily milk production per cow (0.60 kg/cow/day) when the average morning stable fly count was one fly/cow leg after adjusting for parity, THI, study month, and DIM. This reduction could be attributed to stable fly-repelling behaviors, as stable flies are blood-sucking insects that commonly cause painful bites on the lower parts of the body, and cows tend to protect themselves by expressing a bunching behavior [2,12,22]. The loss in milk production per cow due to stable fly attack varied from a minimum of 0.22 kg/cow/day when the mean stable flies are 0.38 fly/cow leg to a maximum of 1.60 kg/cow/day when the mean stable flies are 2.67 fly/cow leg after accounting for the variability in number of stable flies per cow leg per cow pen during the stable fly season (May through July) [4,23,24]. Dairy herds with pens had a mean of stable flies of 1.34 flies/cow leg, which produced, on average, 38.3 kg milk/cow/day in comparison to 39.1 kg/cow/day for herds with zero stable flies/cow leg. The economic loss varied by the study month, with the highest loss estimated during the last week of May, USD 1.86/cow/week (USD 0.27/cow/day), and the lowest was during the last week of July, USD 0.29/cow/week (USD 0.04/cow/day). The average estimated annual loss per cow per year in the Central Valley of California due to stable fly attack was USD 11.76 cow/year, varying between USD 5.41 and USD 18.00/cow/year. For a 1000-cow dairy, the estimated average milk losses due to stable fly attack were, on average, 800 kg/day during the stable fly season (a minimum of 0.22 kg/cow/day to a maximum of 1.60 kg/cow/day). The losses after accounting for the milk price and feed price were USD 11,765 per year. The losses reported in the current study may be underestimated, as the study dairies used different fly control measures that may have influenced the mean stable fly count/pen.

Variations in cow productivity, climate conditions, and currency exchange rates among different regions and different years may influence the economic losses associated with cow bunching and stable flies. To overcome this challenge, the current study modeled the impact of cow bunching and stable fly attack on milk production after accounting for the ambient temperature and relative humidity using the THI, cow parity, and the study month. The losses according to the study models were 0.45 kg of milk if bunching occurred and 0.60 kg of milk when the average stable fly count was one fly/cow leg after accounting for the other confounders. These estimated losses represent nearly 1–1.5% of the daily cow productivity. The inputs of the study models for bunching or fly counts (Table 1 and Table 2) can be utilized by users to compute cow-level milk losses after inputting their respective THI and cow parity. In addition, users in different regions can estimate the economic losses by updating the inputs on the incidence of cow bunching or the mean stable fly count/cow leg with the milk and feed prices, which increases the applicability and generalizability of the current study models. Due to the variation between the dairies in the incidence of cow bunching and the stable fly attack, the study models can help producers estimate their annual losses and decide on their budget to control stable flies and reduce the incidence of cow bunching.

### 4.3. Limitations

The current study relied on data (stable fly activity, cow bunching, and milk) collected from a single Holstein dairy farm in California, which may impact the generalizability of the current results to other dairies with different pen designs, management, breed, and environmental conditions. The current study data on the incidence of cow bunching, mean stable fly count/cow leg, THI, and milk and feed prices were collected during 2017, which might be different than the current values, but our study models can be easily updated with the recent inputs to provide the estimated losses. The economic losses reported in the study may be underestimated, as the study dairies used different fly control methods that might have had an impact on the stable fly population on the dairy and the incidence of cow bunching. More research is needed to address the impact of cow bunching and stable flies on milk production in dairy cows of different breeds under various environmental conditions, different fly control programs, facility designs, and various diets or nutritional supplementation of cows.

## 5. Conclusions

The current study showed a significant reduction in the daily milk production per cow (0.45 kg/cow/day) when bunching occurred, and 0.6 kg/cow/day due to stable fly biting activity. The estimated total loss per cow per year in the Central Valley of California varied between USD 1.35 and USD 4.06, with an average of USD 2.62, for cow bunching, and USD 5.41 to USD 18.00, with an average of USD 11.76, for stable fly attacks. Milk and feed prices are major contributors to the estimation of the economic losses associated with cow bunching. They affect producers’ willingness to invest in controlling stable flies to reduce cow bunching during the fly season. To reduce the economic losses associated with cow bunching and stable fly attacks on dairies, dairy producers may need to focus their efforts on controlling stable flies early during the fly season.

## Figures and Tables

**Table 1:** Results of the two-piece spline mixed model of the association between cow bunching against stable flies and milk production on a California dairy.

Variable	Level	Estimate	Standard Error	*p*-Value	95% CI	
					Lower Limit	Upper Limit
Bunching	No	Reference				
	Yes	−0.45	0.104	<0.01	−0.65	−0.24
Parity	Lactation 1	Reference				
	Lactation 2	5.07	0.400	<0.01	4.28	5.85
	Lactation ≥ 3	7.25	0.412	<0.01	6.45	8.06
Month	July	Reference				
	May	1.09	0.156	<0.01	0.79	1.40
	June	1.72	0.129	<0.01	1.48	1.98
THI	<72	Reference				
	72–78	0.92	0.141	<0.01	0.64	1.19
	>78–89	−0.69	0.086	<0.01	−0.86	−0.52
	>89–98	−4.70	0.148	<0.01	−4.99	−4.41
DIM-Pre-peak		17.33	0.966	<0.01	15.44	19.23
DIM-Post-peak		−12.76	0.422	<0.01	−13.59	−11.93
DIM-Pre-peak square		17.25	1.910	<0.01	13.51	20.99
DIM-Post-peak square		7.60	0.404	<0.01	6.81	8.40
Intercept		40.56	1.500	<0.01	37.62	43.50

**Table 2:** Results of the two-piece spline mixed model of the association between stable fly biting (leg count) and milk production on a California dairy.

Variable	Level	Estimate	Standard Error	*p*-Value	95% CI	
					Lower Limit	Upper Limit
Stable Fly Activity	Stable flies/cow leg	−0.60	0.075	<0.01	−0.75	−0.45
Parity	Lactation 1	Reference				
	Lactation 2	6.32	0.547	<0.01	5.25	7.39
	Lactation ≥ 3	7.70	0.573	<0.01	6.57	8.82
Month	July	Reference				
	May	3.12	0.188	<0.01	2.75	3.49
	June	2.50	0.160	<0.01	2.18	2.81
THI	<72	Reference				
	72–78	1.66	0.174	<0.01	1.32	2.01
	>78–89	−0.80	0.113	<0.01	0.58	1.02
	>89–98	−3.38	0.186	<0.01	−3.74	−3.01
DIM-Pre-peak		28.20	1.149	<0.01	25.95	30.45
DIM-Post-peak		−9.55	0.440	<0.01	−10.42	−8.69
DIM-Pre-peak square		27.38	2.072	<0.01	23.32	31.44
DIM-Post-peak square		5.89	0.395	<0.01	5.12	6.67
Intercept		36.59	2.196	<0.01	32.29	40.90

**Table 3:** Stochastic inputs, their distribution, and parameters used to estimate economic losses associated with cow bunching or stable fly activity.

Variable	Distribution	Parameters
Temperature (°F)	Normal	Average, std = Figure 1
Relative humidity	Normal	Average, std = Figure 1
Percentage of pens that bunch (average per week)	Normal	Average, std = Figure 2
Number of flies (mean leg count per cow)	Pert	Min, most likely, max = Figure 3
Milk price (USD/kg)	Normal	Average = 0.35, std = 0.02
Feed price (USD/kg of milk)	Normal	Average = 0.19, std = 0.01

## Data Availability

The data presented in this study are available upon request from the corresponding author, as the study dairies have not provided consent to publish them alongside the article.
